# Retrospective Enhanced Bat Lyssavirus Surveillance in Germany between 2018–2020

**DOI:** 10.3390/v13081538

**Published:** 2021-08-03

**Authors:** Antonia Klein, Sten Calvelage, Kore Schlottau, Bernd Hoffmann, Elisa Eggerbauer, Thomas Müller, Conrad M. Freuling

**Affiliations:** 1 Friedrich-Loeffler-Institut (FLI), 17493 Greifswald-Insel Riems, Germany; antonia.klein@fli.de; 2 Institute of Diagnostic Virology, Friedrich-Loeffler-Institut (FLI), 17493 Greifswald-Insel Riems, Germany; Sten.Calvelage@fli.de (S.C.); kore.schlottau@fli.de (K.S.); bernd.hoffmann@fli.de (B.H.); 3 Institute of Molecular Virology and Cell Biology, Friedrich-Loeffler-Institut (FLI), WHO Collaborating Centre for Rabies Surveillance and Research, OIE Reference Laboratory for Rabies, 17493 Greifswald-Insel Riems, Germany; Elisaeggerbauer@gmx.de (E.E.); Thomas.Mueller@fli.de (T.M.)

**Keywords:** bat lyssavirus, bat rabies surveillance, European bat lyssavirus 1 (EBLV-1), Bokeloh bat lyssavirus (BBLV), zoonosis

## Abstract

Lyssaviruses are the causative agents for rabies, a zoonotic and fatal disease. Bats are the ancestral reservoir host for lyssaviruses, and at least three different lyssaviruses have been found in bats from Germany. Across Europe, novel lyssaviruses were identified in bats recently and occasional spillover infections in other mammals and human cases highlight their public health relevance. Here, we report the results from an enhanced passive bat rabies surveillance that encompasses samples without human contact that would not be tested under routine conditions. To this end, 1236 bat brain samples obtained between 2018 and 2020 were screened for lyssaviruses via several RT-qPCR assays. European bat lyssavirus type 1 (EBLV-1) was dominant, with 15 positives exclusively found in serotine bats (*Eptesicus serotinus*) from northern Germany. Additionally, when an archived set of bat samples that had tested negative for rabies by the FAT were screened in the process of assay validation, four samples tested EBLV-1 positive, including two detected in *Pipistrellus pipistrellus*. Subsequent phylogenetic analysis of 17 full genomes assigned all except one of these viruses to the A1 cluster of the EBLV-1a sub-lineage. Furthermore, we report here another Bokeloh bat lyssavirus (BBLV) infection in a Natterer’s bat (*Myotis nattereri*) found in Lower Saxony, the tenth reported case of this novel bat lyssavirus.

## 1. Introduction

Bats (Chiroptera) have been identified or were suspected of being reservoir hosts for a plethora of viruses including those with a zoonotic potential [1]. Among the latter, there are pathogens of high concern like Ebolaviruses, Henipaviruses, Coronaviruses and Lyssaviruses [2,3,4,5,6]. Interestingly, rabies is the oldest known bat associated infection in humans. Rabies in bats was first identified in the Americas, but has ever since been found on all continents except Antarctica [7]. The causative agents are different lyssaviruses of the family *Rhabdoviridae* within the order *Mononegavirales* [8]. Of note, with two exceptions, all of the 18 known lyssaviruses are associated with bats, their assumed ancestral primary reservoir hosts [9]. 

Six distinct lyssaviruses have been isolated from European bats, whereby the majority of reported cases is caused by European bat lyssavirus type 1 (EBLV-1) [10]. European bat lyssavirus type 2 (EBLV-2) was identified in only two dozen cases [11], while West Caucasian bat lyssavirus (WCBV) and Lleida bat lyssavirus (LLBV) were only isolated sporadically. Bokeloh bat lyssavirus (BBLV) [12] and Kotalahti bat lyssavirus (KBLV) [13] further extended the diversity of lyssaviruses found in European bats. 

EBLV-1, EBLV-2 and BBLV are presently known to circulate in bats in Germany [10]. EBLV-1 caused the majority of the 346 reported German bat rabies cases [14], and has mainly been associated with the serotine bat (*Eptesicus serotinus*) [15]. In contrast, EBLV-2 was only isolated five times from Daubenton’s bat (*Myotis daubentonii*) [11]. BBLV was first discovered in a Natterer’s bat (*Myotis nattereri*) in Lower Saxony in 2010 [12]. Ever since, it has been isolated several times from this species in Germany, France and Poland [16,17], suggesting that the Natterer’s bat is the reservoir host species. 

All bat lyssaviruses are potentially capable of infecting other mammals including humans and cause the fatal disease rabies. In fact, in Europe bat lyssaviruses were identified in spillover infections in cats [18], sheep [19], and a stone marten [20]. Also, human rabies cases caused by EBLV-1 [21,22] and EBLV-2 [23,24] infections were confirmed, thus highlighting the zoonotic potential and public health importance. Therefore, to advance our understanding on epidemiology of bat-related lyssaviruses, surveillance activities are ongoing across Europe [15]. Against this background, we here report recent results from an enhanced passive surveillance scheme in Germany. By using molecular methods as opposed to previously applied fluorescent antibody test (FAT), we could detect several EBLV-1 cases, including two in the common pipistrelle (*Pipistrellus pipistrellus*). Additionally, we identified the tenth case of BBLV in a Natterer’s bat.

## 2. Materials and Methods

### 2.1. Bat Samples

Dead found bats are regularly collected by local bat biologists, private bat handlers, different wildlife care centers as well as nature conservation institutions, and are stored under frozen conditions. Upon request and by providing cool boxes for shipment, bats were submitted to the Friedrich-Loeffler-Institute (FLI) for lyssavirus diagnosis. Information regarding bat species, geographical origin, and sex, was provided by the bat handlers. Where information on the bat species was missing, bats were determined to genus or species level by external morphological characteristics [25,26]. A total of 117 individuals could not be clearly specified due to a decomposed condition. 

Additionally, an archived set of bat samples that had been tested negative for rabies by the FAT [15] was screened in the process of assay validation. 

### 2.2. Brain Sample Generation

Brain tissue from bats was sampled by puncturing the *foramen occipitale magnum* using a syringe and a 0.90 × 40 mm cannula. Initially, brain tissue was aspirated and flushed out into Eppendorf vials using cell culture media. Then the cranial cavity was repeatedly flushed with cell culture media and aspirated. Here, a mixture of equal volumes of Eagle MEM (Hanks’ balanced salts solution) and Eagle MEM (Earle’s balanced salts solution) medium, supplemented with 10% fetal bovine serum was used. Extracted brain tissue was homogenized in a volume of 1000 µL cell culture media and stored at −80 °C until further testing.

### 2.3. RNA Extraction

Total RNA was extracted from 100 µL of brain suspension in a BioSprint 96 magnetic particle processor (Qiagen, Hilden, Germany) using the NucleoMagVet kit (Macherey & Nagel, Düren, Germany) according to the manufacturer’s instructions. A final volume of 100 µL nucleic acid was generated.

### 2.4. Virus Detection by RT-qPCR

For the detection of lyssaviral RNA, a double-check approach was used [27]. On the one hand, a pan-lyssa real-time RT-PCR targeting both the N- and L-gene with Resolight as intercalating dye was conducted. The RT-PCR reaction was prepared using the OneStep RT-PCR kit (Qiagen), adjusted to a volume of 12.5 µL, with 2.5 µL of extracted nucleic acid added. The reaction included 10 min at 45 °C for reverse transcription and 10 min at 95 °C for activation, followed by 45 cycles of 15 s at 95 °C for denaturation, 20 s at 56 °C for annealing and 30 s at 72 °C for elongation, respectively.

To specifically detect RNA of EBLV-1, EBLV-2 and BBLV a modification of the R14-assay [27], i.e., the RABV probe was omitted, the EBLV-1 probe was FAM-labelled, and ß-Actin-mix2-HEX was included as internal control assay. To this end, the AgPath-ID One-Step RT-PCR kit (Thermo Fisher Scientific, Waltham, MA, USA) was applied in a total volume of 12.5 µL including 2.5 µL of beforehand extracted nucleic acid were added to 10 µL of the master mix [27]. 

All RT-qPCRs were run on a BioRad CFX96 Real-Time System (Bio-Rad, Hercules, CA, USA). Negative (RNA isolation control, no template control) and positive (EBLV-1, EBLV-2, BBLV) controls were analyzed in parallel with each PCR run. 

### 2.5. Virus Isolation in Cell Culture

Virus isolation was attempted for all brain suspension samples that had initially been tested positive for lyssaviral RNA using the rabies tissue-culture infection test (RTCIT) as described before [28]. Briefly, bat brain suspensions were centrifuged and 500 µL supernatant was equally mixed with 10^6^ mouse neuroblastoma cells (NA42/43; CCLV-RIE 0229, Collection of Cell Lines in Veterinary Medicine (CCLV) at the FLI, Riems) and incubated at 37 °C with 5% CO_2_ for 30 min. Cells were maintained in a mixture of equal volumes of Eagle MEM (Hanks’ balanced salts solution) and Eagle MEM (Earle’s balanced salts solution) medium, and supplemented with 10% fetal bovine serum and 1% Pen-Strep (10,000 U/mL).

After further centrifugation, cell pellets were resuspended in T25 cell culture flasks and incubated for three to four days under the same conditions as stated above. Additionally, a control dish was set up in parallel for each passage. After three to four days the control was fixed, stained with a fluorescein isothiocyanate (FITC) conjugated polyclonal antibody (SIFIN, Berlin, Germany), washed and checked for the presence of virus. If viral antigen was detected, the test result was declared positive. A sample was considered negative after three consecutive serial passages without viral growth.

### 2.6. NGS Sample Processing

Preparation of Ion Torrent compatible sequencing libraries was conducted according to an adapted version of the NGS-based metagenomics pathogen detection workflow published by Wylezich et al. [29]. In short, homogenized brain material was combined with 1 mL Trizol and subsequently treated with chloroform. For sample 45369, a mixture of 250 µL cell culture supernatant and 750 µL Trizol LS was used instead. After centrifugation, 400 µL of the aqueous phase was used for RNA extraction on a KingFisher Flex platform (Thermo Fisher Scientific, Waltham, MA, USA) in combination with the RNAdvance Tissue Kit (Beckman Coulter, Brea, CA, USA) and included DNase I digestion step. Double stranded cDNA was generated from 350 ng total RNA under usage of the SuperScript™ IV First-Strand cDNA Synthesis System (Invitrogen/Thermo Fisher Scientific, Waltham, MA, USA) and the NEBNext^®^ Ultra™ II Non-Directional RNA Second Strand Synthesis Module (New England Biolabs, Ipswich, MA, USA). After ultrasonic fragmentation on a Covaris M220 (Covaris, Brighton, UK), ds cDNA was converted to Ion Torrent compatible libraries utilizing the GeneRead L Core Kit (Qiagen) in combination with IonXpress barcode adaptors (Thermo Fischer Scientific) followed by a size selection step targeting for library fragments of approx. 500 bp size. Sample processing steps related to cDNA generation, library preparation and size selection were conducted on a Biomek 4000 automated liquid handler (Beckman Coulter). Subsequently, sequencing libraries were quality controlled (2100 Bioanalyzer, High sensitivity DNA Kit, Agilent Technologies, Santa Clara, CA, USA) and quantified (QIAseq Library Quant Assay Kit, Qiagen) to ensure optimal sequencing results. Libraries were sequenced on an Ion Torrent S5XL instrument (Thermo Fisher Scientific) utilizing Ion 530 chips and reagents according to the manufacturer’s instructions. 

Processing of samples 5668, 31955, 23549 and 23157 was adjusted considering the highly decomposed state of the original sample material. For these samples, RNA extraction was conducted utilizing the RNeasy Mini Kit (Qiagen) and an on-column DNase I digestion step. Subsequently, cDNA was generated using the cDNA synthesis system kit (Roche Diagnostic, Rotkreuz, Switzerland) in combination with random hexamer primers (Roche Diagnostic). After library preparation, small library fragments (~200 bp size) were separated from standard size fragments (~500 bp) in the size selection step and kept for further processing instead of being discarded. Standard libraries (500 bp) derived from samples 5668 and 23157 as well as small fragments (200 bp) of sample 23157 were amplified using the GeneRead DNA Amp L Kit (Qiagen). Amplified libraries were purified twice with a 1.2× volume of Agencourt AMPure XP beads (Beckman Coulter) to remove any interfering substances and remaining adapter dimers. Sequencing of small fragment libraries was realized on Ion 540 chips and reagents according to the manufacturer’s instructions. 

### 2.7. Generation of Full Genome Sequences and Phylogenetic Analysis

Raw sequencing data were automatically adapter trimmed by the Ion Torrent Software Suite (v.5.12.1) and subsequently mapped against the EBLV-1 reference sequence (NC_009527) using the 454 Sequencing System Software v3.0 (Roche). Full genome sequences were obtained by de novo assembly of full or partial mapped reads and annotated with Geneious Prime (2021.0.1, build 2020-12-01). Phylogenetic analyses were conducted with IQ-TREE (v. 1.6.5) under usage of the ultrafast bootstrap approximation approach (100.000 ultrafast bootstrap) and enabled ModelFinder feature [30] for maximum-likelihood phylogenetic tree construction (best-fit model: GTR+F+R2). Therefore, a dataset of 127 EBLV-1 full genome sequences was investigated encompassing the newly generated German EBLV-1 full genome sequences and 111 sequences from previously published datasets. German cases with only partial genomes were excluded from phylogenetic analysis. 

## 3. Results

### General Surveillance

During a period of 30 months a total of 1236 bats were sampled and investigated under this scheme, comprising of animals that have been collected within and before this study period, with the oldest sample originating from 2004. Samples were received from ten different participating German federal states, with the majority of dead bats originating from Lower Saxony (*N* = 464), followed by Berlin (*N* = 252) and Baden-Wuerttemberg (*N* = 167). The sample set encompassed 18 different bat species from the family *Vespertilionidae*, with the *Pipistrellus pipistrellus* (*N* = 625) being the most frequently sampled bat species, followed by *Eptesicus serotinus* (*N* = 96) and *Nyctalus noctula* (*N* = 89) (Table 1). For 9.5% of all bats (*N* = 117) the species could not be determined. Of all analyzed bats with known gender, 54% were male and 46% were female.

In total, 16 samples tested positive for lyssaviral RNA by RT-qPCR (Table 1 and Table 2). All those specimens tested positive in the N-gene pan-lyssa PCR, and were confirmed to the virus species level by the specific R14 RT-qPCR assays. Virus isolation was not successful in two cases and sequencing data received from one of those samples were insufficient to obtain a virus genome sequence. 

**Figure 1:**
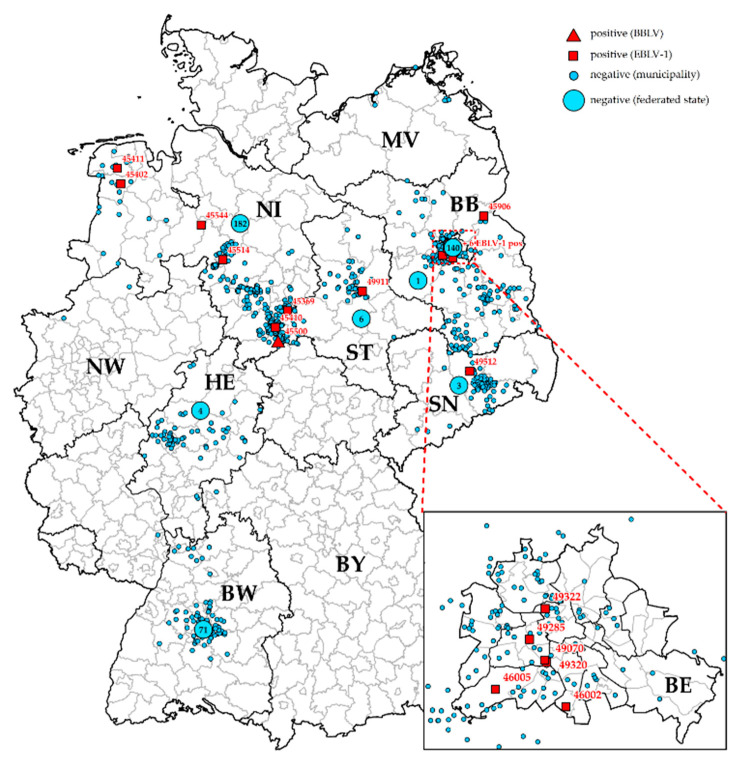
Spatial distribution of analyzed bat specimen, with positive cases indicated (red). Numbers in larger circles correspond to specimen for which detailed information on the origin was not available. Red dashed box: area of Berlin enlarged to visualize the distribution of samples. Abbreviations for German federal states: Baden-Wuerttemberg (BW), Bavaria (BY), Berlin (BE), Brandenburg (BB), Hesse (HE), Lower Saxony (NI), Mecklenburg-Western Pomerania (MV), Northrhine-Westphalia (NW), Saxony-Anhalt (ST), Saxony (SN).

The vast majority of positive specimens was found in bats from Lower Saxony (*N* = 7) and Berlin (*N* = 6), in contrast to only one lyssavirus infection detected in Brandenburg, Saxony-Anhalt and Saxony, respectively (Figure 1). Despite a relatively high number of submitted animals, no lyssaviral RNA was detected in bats from Baden-Wuertemberg in the ongoing study (Figure 1, Table 1), but in samples that were screened retrospectively (Table 2). Viruses characterized as EBLV-1 were predominately detected in serotine bats, and in two common pipistrelles (Table 1 and Table 2).

**Figure 2:**
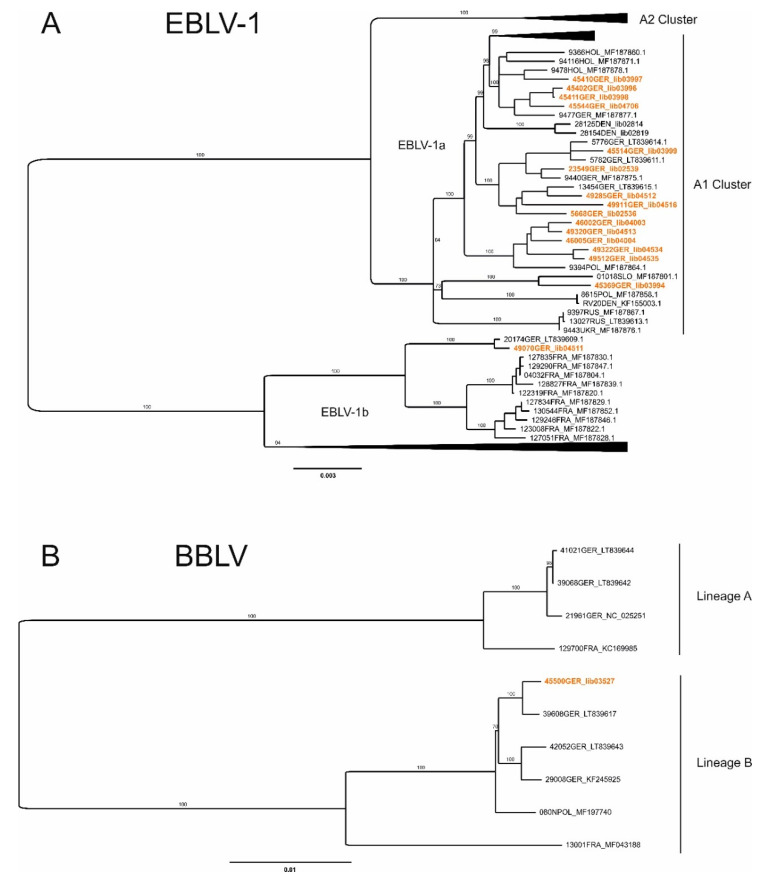
Mid-point rooted maximum-likelihood phylogenetic trees showing (**A**) the genetic diversity of 127 EBLV-1 full genome sequences originating from nine different countries, and (**B**) all available full genome sequences for BBLV. All newly generated full genome sequences (see Table 2) are indicated (orange).

PCR-positive samples were subjected to next generation sequencing, resulting in the generation of complete/nearly complete genome sequences for most samples (Table 2). Subsequent phylogenetic analyses of the newly generated German EBLV-1 sequences revealed the grouping of the majority of the investigated cases within the A1 cluster of the EBLV-1a sub-lineage (Figure 2A), as proposed [31], and exhibited a sequence identity of 98.7% between the 15 considered German sequences. Furthermore, these cases were represented in three distinct phylogenetic groups within the A1 cluster. Nearly exclusively formed by German isolates, the first group included five of the newly generated EBLV-1 sequences (sample 46002, 49320, 46005, 49322 and 49512) distributed over the eastern part of Germany (Berlin, Saxony) as well as a single Polish EBLV-1 case. A second group encompassed sequences of new and already published German cases that were mainly found in central regions of the country (samples 5668, 23549, 49285, 49911 and 45514). Lastly, a third group of German and Dutch cases was extended by four new German EBLV-1 viruses (sample 45410, 45402, 45411 and 45544) from areas near the German-Dutch border. Interestingly, despite its geographic location in central Germany, sample 45369 was separated from other German cases and clustered closely with a Slovakian EBLV-1a sequence. Besides EBLV-1a, one of the investigated cases (sample 49070) was identified as member of the EBLV-1b sub-lineage, clustering closely with a previously found German EBLV-1b case from the year 2008 from Halle/Saale (20174GER, Figure 2A).

The single bat that tested BBLV positive was found dead in the area of Herzberg, District of Göttingen, in southern Lower Saxony (GPS-coordinates: 51°39′5.218″ N/10°20′16.687″ E) and was identified as a female Natterer’s bat. Full genome sequencing revealed 99.7% sequence identity with a BBLV case detected earlier in Kronach, Bavaria in 2015 [17]. The close genetic relationship is illustrated in Figure 2B.

## 4. Discussion

This study provides novel insight into the epidemiology of bat-related lyssaviruses in Germany. To this end, more than 1000 bats were sampled and analyzed over a period of two and a half years, yielding results comparable to previous studies with similar focus [10,15]. In Germany, routine bat rabies surveillance performed by regional veterinary laboratories is focused on bats associated to human contact or which show signs of clinical disease suggestive of rabies [33]. While this surveillance scheme is important for the immediate public health intervention, it is inherently biased. Therefore, this sample set should be complemented by enhanced passive surveillance, i.e., the integration of dead found bats without human contact (e.g., found in caves, forests, etc.), as recommended before [15,34]. This allows for a higher sampling intensity and provides a better picture of the occurrence and distribution of bat lyssaviruses. In our study, we supported submissions by providing shipment material and covering the costs for transportation. Also, by non-destructive sampling outside the BSL-3 facility, we could offer to return bats that tested negative. These facts may have led to higher willingness of bat handlers for sample submissions. Unfortunately, this scheme could not be applied uniformly across Germany, as can be seen from the origin of the submissions (Figure 1). The practical implementation was hampered by different constraints on various levels, including, e.g., the heterogeneous landscape of bat conservation in Germany, and different regulations in federal states on the conservation and archival of endangered and protected species.

The number of submitted individuals per bat species varied, ranging from one animal (*Myotis bechsteinii*) to 625 (*Pipistrellus pipistrellus*). This variation may be reflective of population numbers of particular bat species, which, however, are difficult to estimate. Similar to previous surveillance studies from Europe [15,35,36,37], the common pipistrelle was the most frequently submitted bat species, which is consistent with the fact that it is one of the most abundant synanthropic European bat species [26]. Taken together, the results of our study need to be carefully assessed and should not be considered representative for the respective bat species.

By investigating dead found bats the animals can also be screened for other pathogens and viruses besides lyssaviruses including, for example, Coronaviruses. Recent findings of novel lyssaviruses, e.g., KBLV in Finland [13] and Matlo bat lyssavirus (MBLV) in South Africa [38], confirm the necessity for such surveillance studies. While negative results do not exclude the presence of lyssaviruses in the bat population, positive samples and isolated viruses thereof are essential for further characterizations, including phylogenetics, pathogenesis in animal models and cross-neutralization by available vaccines. This contributes to a risk assessment for novel bat lyssaviruses, as exemplified for BBLV [39], LLBV [40] and KBLV [41].

Historically, the FAT was regarded as the gold standard in rabies diagnostics but recently recommendations by both the WHO and OIE were updated, allowing the use of RT-qPCR as a primary diagnostic test since it also demonstrates a very high diagnostic sensitivity and specificity [42]. Consequently, we changed the previous screening strategy for bat-associated lyssaviruses to using different RT-qPCR assays for a more convenient and therefore faster analysis. Our approach for non-destructive sampling was based on previous recommendations for surveillance in larger mammals [43]. Due to the fact that the bat carcasses were often in a state of decomposition and subject to freeze-thawing, bat brains were mostly liquefied. Therefore, aspiration was easily performed, and in fact, sufficient material could be obtained as visually checked and confirmed by beta-actin results of the PCR. If the diagnostic sensitivity was lowered by the sampling technique, which cannot be completely ruled out, this is outweighed by the increased submissions and sensitivity of the molecular techniques used. 

Screening each sample in a double-check approach allows a diagnostic maximum in finding known and potentially novel lyssavirus species. Especially in bat surveillance, working with poor quality samples and additionally very small amounts is a common case, where molecular methods offer a higher sensitivity [44]. This is exemplified by the additional EBLV-1 cases identified in samples (Table 2) that initially tested FAT-negative in a previous retrospective study [15].

The predominance of EBLV-1 (94% of all positive bats) corroborate results of previous bat rabies surveillance studies [15,45]. Also, the positivity rate of 16% in serotine bats is comparable to results observed in a previous German enhanced passive surveillance study, where 13% of all tested serotine bats were found to be EBLV-1-positive [15]. Similarly, in Spain (*Eptesicus isabellinus*) and the Netherlands a positivity rate for EBLV-1 of about 20% was reported [34,46]. Spillover infections of EBLV-1 to bat species other than *E. serotinus* and *E. isabellinus* are rarely found [10,15,47,48]. Spillover infections of EBLV-1 into bats other than *E. serotinus* could not be detected in the submitted samples between 2018−2020. However, screening of a large number of bat samples that had initially tested negative by FAT [15] by using molecular methods revealed two cases in common pipistrelles (Table 2). Interestingly, those bats were found in the southeastern federal state of Baden-Wurttemberg, a region without known cases of EBLV-1. The results demonstrate that spillover events can also be observed in regions with hitherto undetected occurrence of EBLV-1. 

The spatial distribution of EBLV-1 positive bats generally confirmed previous patterns of distribution, with the majority of cases found in the North of Germany [15,32]. This was explained by higher population density of serotine bats in this region which seems to support the intraspecies transmission and virus maintenance [32]. Interestingly, six positive cases were detected in the urban area of Berlin. This apparent aggregation of cases is likely biased by the fact that the number of submitted samples from this area was very high. Whether this is due to higher abundance of the serotine bat, or the increased encounters of bats by members of the public, which is likely for this synanthropic bat species, is arguable [49].

Genetically, all except one EBLV-1 isolate can be assigned to sub-lineage EBLV-1a, which is considered to exhibit a relatively higher genetic homogeneity compared to EBLV-1b [50]. However, a higher phylogeographic segregation of EBLV-1a sequences with the A1 cluster can be observed, similar to a recent analysis on Danish EBVL-1 samples [48]. EBLV-1b occurrence in Germany is centered in the west, close to the border with France [15]. Here, we report an additional EBLV-1b case in the eastern part of Germany, which supports the assumption that this sub-lineage is distributed beyond its known expansion in western European countries like Spain, France and the Netherlands [31]. 

Within our study, we identified the tenth BBLV case, which is the seventh case in Germany and the fourth case in Lower Saxony isolated from a Natterer’s bat. Since its first detection in 2010 in Germany [12], BBLV was found several times in Germany, France and Poland [16,17]. The fact that it was again isolated from the same bat species supports the hypothesis of the Natterer’s bat representing the reservoir host species. 

Interestingly, the BBLV from Herzberg in Lower Saxony is genetically closer related to BBLV detected in Kronach, Bavaria in 2015 [51] than to other cases found in Lower Saxony. The isolate from Kronach is again closely related to an isolate from Poland [16]. The apparent discrepancies between phylogenetic grouping and geographic origin are difficult to explain [17]. Also, the fact that BBLV has only recently been discovered but ever since appears to be more prevalent than, for example, EBLV-2, is puzzling and cannot be explained by increased surveillance activities. Further investigations would be needed to elucidate these phenomena. 

## 5. Conclusions

Bat rabies surveillance is only operative where dedicated people involved in bat conservation, biology, research, etc., are working together with veterinary scientists in a true One Health approach. Without their additional effort and motivation, such studies would not be feasible, and we would like to reiterate our acknowledgement to all parties and numerous individuals that contributed bat specimen. 

While taking the limitations of passive surveillance data into account, nonetheless it is essential for the identification of known and novel pathogens, as exemplified by the discovery of BBLV [12] and KBLV [13]. 

The results of our study support that enhanced passive bat rabies surveillance can gain sensitivity by applying RT-qPCR screening. The methodology is also more convenient and could offer a higher throughput. We therefore recommend a nationwide and eventually European enhanced passive surveillance via RT-qPCR-screening complementary to testing suspected bats with human contact. 

Biased sampling, as in this enhanced passive surveillance scheme, cannot fully reflect the true prevalence and the correct epidemiological bat rabies situation. The 1.2% positivity across all species is similar to values found in retrospective studies in France [52]. While this value may appear to be of a relatively low level, rabies in bats poses a potential veterinary and public health risk. This risk is especially eminent for people handling bats for research or conservation reasons. Mitigating measures should include preventing bites by, e.g., using gloves and adequate pre- and post-exposure prophylactic treatments according to international and national guidelines. 

## Figures and Tables

**Table 1:** Details of passive bat rabies surveillance. Numbers of animals investigated per species and federal state. Lyssavirus-positive cases are indicated (in brackets). All viruses were characterized as EBLV-1, except for one case in the Natterer’s bat. Abbreviations for German federal states: Baden-Wuerttemberg (BW), Bavaria (BY), Berlin (BE), Brandenburg (BB), Hesse (HE), Lower Saxony (NI), Mecklenburg-Western Pomerania (MV), Northrhine-Westphalia (NW), Saxony-Anhalt (ST), Saxony (SN).

Species	BB	BE	BW	BY	HE	MV	NI	NW	SN	ST	Total
*Barbastella barbastellus*	1						1		1		3
*Eptesicus nilssonii*							3				3
*Eptesicus serotinus*	22 (1)	35 (6)	1		2		22 (6)		5 (1)	9(1)	96 (15)
*Myotis* ssp.							7				7
*Myotis bechsteinii*			1								1
*Myotis brandtii*					3		28				31
*Myotis daubentonii*	4	2	2				18		4	3	33
*Myotis myotis*	3	1	3		1		2		2		12
*Myotis mystacinus*	2		8		1		32		2		45
*Myotis nattereri*	8	4			1		12 (1) ^#^		2	3	31 (1)
*Nyctalus* ssp.										2	2
*Nyctalus leisleri*		2			11		2		1		16
*Nyctalus noctula*	22	35	1			5	11	2	13		89
*Pipistrellus kuhlii*	1					1					2
*Pipistrellus nathusii*	7	2	9		1		13		5	1	38
*Pipistrellus pipistrellus*	52	152	73	1	44	2	251		28	22	625
*Pipistrellus pygmaeus*	10						3		1	1	15
*Plecotus* ssp.							1			1	2
*Plecotus auritus*	4	1	3		1		15		3	4	31
*Plecotus austriacus*	2	1			1		2				6
*Vespertilio murinus*	3	9	2		1		8		7	1	31
unspecified	4	8	64		5		32		1	3	117
**total**	**145 (1)**	**252 (6)**	**167**	**1**	**72**	**8**	**463 (7)**	**2**	**75 (1)**	**50 (1)**	**1236 (16)**

# Characterized as BBLV.

**Table 2:** Details for bat samples that tested positive for EBLV-1 and BBLV. Cq-Values for the different RT-PCR assays are provided. For sample 45906, NGS only generated two viral reads and the lyssavirus species was determined with PanLyssavirus hn-RT-PCR [32] and subsequent Sanger sequencing. Abbreviations for German federal states: Baden-Wuerttemberg (BW), Berlin (BE), Brandenburg (BB), Lower Saxony (NI), Mecklenburg-Western Pomerania (MV), Saxony-Anhalt (ST), Saxony (SN).

Lab-ID	Collection Date	Host	Sex	Location	Virus	R14 EBLV-1	R14 EBLV-2	R14 BBLV	Pan-N	Pan-L	Virus Isolation	Library Number	Genome Length	Sequence
45402	July 18	*E. serotinus*	f	Holtland (NI)	EBLV-1a	13.84	-	-	30.95	36.19	+	Lib03996	11,962	Nearly Complete
45410	n.a.	*E. serotinus*	m	n.a. (NI)	EBLV-1a	14.45	-	-	26.85	32.19	+	Lib03997	11,953	Nearly Complete
45411	May 16	*E. serotinus*	m	Westerende Holzloog (NI)	EBLV-1a	15.29	-	-	31.72	39.62	+	Lib03998	11,963	Nearly Complete
45514	August 16	*E. serotinus*	m	Wunstorf (NI)	EBLV-1a	9.17	-	-	43.60	40.03	+	Lib03999	11,965	Nearly Complete
45544	August 12	*E. serotinus*	m	Bruchausen-Vilsen (NI)	EBLV-1a	9.77	-	-	39.18	32.39	+	Lib04706	11,966	Complete
46002	August 18	*E. serotinus*	f	Berlin-Marienfelde (BE)	EBLV-1a	14.76	-	-	35.95	37.02	+	Lib04003	11,966	Complete
46005	May 18	*E. serotinus*	m	Berlin-Nikolassee (BE)	EBLV-1a	17.92	-	-	34.68	37.51	+	Lib04004	11,962	Nearly Complete
49070	August 19	*E. serotinus*	f	Berlin-Friedenau (BE)	EBLV-1b	12.02	-	-	27.10	37.05	+	Lib04511	11,967	Complete
49285	September 19	*E. serotinus*	m	Berlin-Witzleben (BE)	EBLV-1a	12.59	-	-	27.40	28.47	+	Lib04512	11,966	Complete
49320	2017	*E. serotinus*	m	Berlin-Friedenau (BE)	EBLV-1a	15.05	-	-	27.70	30.01	+	Lib04513	11,967	Complete
49911	June 19	*E. serotinus*	m	Gommern (ST)	EBLV-1a	12.03	-	-	28.74	29.79	+	Lib04516	11,958	Nearly Complete
49322	2017	*E. serotinus*	f	Berlin-Wedding (BE)	EBLV-1a	9.65	-	-	27.75	28.75	+	Lib04534	11,966	Complete
49512	August 17	*E. serotinus*	f	Niederau (SN)	EBLV-1a	12.07	-	-	28.18	31.51	-	Lib04535	11,964	Nearly Complete
45369	May 16	*E. serotinus*	f	Göttingerode (NI)	EBLV-1a	20.68	-	-	35.51	37.45	+	Lib03994	11,965	Nearly Complete
45906	September 04	*E. serotinus*	f	Falkenberg (BB)	EBLV-1a	24.79	-	-	36.28	-	-	n.a.	n.a.	n.a.
45500	June 14	*M. nattereri*	f	Herzberg (NI)	BBLV	-	-	12.75	44.21	30.06	+	Lib03527	11,896	Nearly Complete
5668	August 2000	*E. serotinus*	n.a.	Kubbelkow (MV)	EBLV-1a	28.65	-	-	33.52	-	+	Lib02536	11,966	Complete
31955	June 12	*P. pipistrellus*	m	Tübingen (BW)	EBLV-1a	-	-	-	32.83	-	+	Lib02538	n.a.	Partial
23549	June 07	*E. serotinus*	m	Halle/Saale (ST)	EBLV-1a	26.92	-	-	29.89	34.1	+	Lib02539	11,966	Complete
23157	April 10	*P. pipistrellus*	m	Nürtingen (BW)	EBLV-1a	-	-	-	34.66	-	+	Lib02580	n.a.	Partial

## Data Availability

All generated full-genome sequences were submitted to the European Nucleotide Archive (ENA) under the study accession PRJEB46019.
